# Lateral Geniculate Nucleus Volume Assessed by 7 Tesla MRI 3D MT-Weighted SILENT Protocol in Patients with STARGARDT Disease—Pilot Study

**DOI:** 10.3390/jcm14165666

**Published:** 2025-08-11

**Authors:** Agata Szpringer-Wabicz, Katarzyna Nowomiejska, Anna Niedziałek, Michał Toborek, Katarzyna Wiśniewska, Mateusz Midura, Mark Symms, Robert Rejdak, Radosław Pietura

**Affiliations:** 1 Chair and Department of General and Pediatric Ophthalmology, Medical University of Lublin, 20-079 Lublin, Poland; agatasara@gmail.com (A.S.-W.); robertrejdak@yahoo.com (R.R.); 2 Department of Radiography, Medical University of Lublin, 20-079 Lublin, Poland; anianiedzialek@interia.pl (A.N.); toborekmichal@gmail.com (M.T.); katarzyna.agnieszka.wisniewska@gmail.com (K.W.); mark.symms@gmail.com (M.S.); radoslawpietura@gmail.com (R.P.); 3 Faculty of Electronics and Information Technology, Institute of Radioelectronics and Multimedia Technology, Warsaw University of Technology, 20-079 Lublin, Poland; mateusz.midura@pw.edu.pl

**Keywords:** Stargardt disease, 7 Tesla MRI, lateral geniculate nucleus

## Abstract

**Background/Objectives:** To quantitatively assess lateral geniculate nucleus (LGN) volume using 7 Tesla MRI in patients with Stargardt disease (STGD). **Methods:** A total of 18 patients with STGD and 15 healthy volunteers were examined with a 7 Tesla MRI of the brain. Measures of LGN volume were performed manually by three independent investigators (radiologists) using ITK-SNAP software, version 4.0.0-rc.2. The volume of the thalamus was evaluated using the open-source automated software package FreeSurfer. Before 7 Tesla MRI, patients underwent ophthalmic examination and 1.5 Tesla MRI. **Results:** The average LGN volume in both hemispheres was significantly smaller in patients with STGD (right, −111.2 mm^3^; left, 107.4 mm^3^) than in the control group (right, −128.7 mm^3^; left, 123.6 mm^3^, respectively) (*p* < 0.0001). The ratio of LGN to thalamus in the right hemisphere was significantly lower (*p* = 0.024) in the group of patients with STGD (0.014) than in the control group (0.017). **Conclusions:** The right and left LGN volumes in MR 7T imaging, as well as the right LGN/thalamus ratio, were reduced in patients with STGD compared to controls. 7T MRI using the 3D MT-weighted SILENT protocol provides new insight into structural changes in the brain in retinal dystrophies and offers a possible marker of the response to future therapies in STGD.

## 1. Introduction

Stargardt disease (STGD) is an autosomal recessive retinal dystrophy whose hallmarks are bilateral progressive central vision loss and subretinal deposition of lipofuscin-like substances [1]. STGD is the most prevalent inherited macular dystrophy and is associated with disease-causing sequence variants in the gene ABCA4 [2]. ABCA4 is the source of biallelic mutations that disrupt the normal function of retinal pigment epithelium cells in the retina [3]. With RPE cell degeneration, there is a failure to support the overlying photoreceptors, leading to the characteristic central vision loss seen in STGD [4].

STGD progresses slowly during the lifespan, and its prevalence is about 1:8000–10,000 [5]. Onset usually occurs in childhood or early adolescence, affecting both males and females [5]. Patients present with evolving, painless, bilateral central vision impairment, with visual acuity between 20/20 and 20/400, as well as a well-circumscribed central scotoma [6]. Characteristic macular atrophy and yellow–white flecks at the level of the retinal pigment epithelium (RPE) at the posterior pole [2] can be seen during fundoscopy. In its advanced stages, patients affected by this hereditary cone–rod dystrophy end up losing macular vision and can only rely on residual peripheral vision in their daily lives. It has an impact on quality of life and places a burden on affected individuals and their families [7]. Moreover, vision loss in STGD can lead to difficulties in reading, performing everyday tasks, and recognizing faces, which can affect educational and occupational opportunities. STGD affects young individuals early in life, and thus they may require long-term support and rehabilitation services.

The lateral geniculate nucleus (LGN) is a small, bilateral structure of the thalamus that receives input from each eye, representing the contralateral half of the visual field. It serves as a relay center from the retina to the primary visual cortex [8]. The LGN is located superior to the hippocampus and medial to the optic radiation [9]. Receptive fields in the LGN are similar to those in the retinal ganglion cell layer in terms of both spatial and temporal characteristics. The structure of the LGN consists of six laminae with associated interlaminar structures that macroscopically segregate the magno-, parvo-, and koniocellular visual streams originating in the anatomically ipsi- and contralateral eyes. The specific contributions of the retina, thalamus, and visual cortex to LGN properties are presently unknown [10]. Studies in animals, such as rodents and monkeys, have shown that the LGN should be considered not just a visual relay center but a key member of the visual system with substantial experience-dependent plasticity [11,12]. We already know that the extent of retinal damage common in large central scotoma should influence the cortical magnification factor for the human foveal retina [13], and a much bigger area of cortex should be affected than in peripheral visual field loss in retinitis pigmentosa.

Inherited retinal dystrophies are progressive in nature, with not only the retina but also downstream visual pathways deteriorating over time [13,14,15]. Over the past few years, research on Stargardt disease has advanced significantly, focusing mostly on clinical features and molecular genetics. However, little is known about how degenerative diseases of the outer retina affect the balance of visual processing in the visual pathway. The LGN was previously difficult to obtain quantitative measurements of in 1.5T and 3T MRI, as in these methods there was partial volume uncertainty, low resolution, and insufficient accuracy for exact LGN visualization and volume quantification. High signal-to-noise ratio (SNR) values in 7 Tesla MRI allow the visualization of tiny thalamic nuclei such as the LGN with higher sensitivity, higher contrast, and better spatial resolution, in comparison to 3 Tesla MRI [16,17]. It is possible to increase LGN visibility in standard MRI by optimizing MR sequence parameters, for example by using white-matter-nulled sequences [18] to enhance gray matter structures, or by increasing CNR through averaging over repetition time [19].

Nowadays, thanks to 7T high-field MRI, the LGN can be assessed in diseases of the visual pathway, including inherited retinal dystrophies. We propose a 7 Tesla MRI examination protocol that allows volumetric analysis of the LGN despite its small size.

Therefore, the aim of the present study was the quantitative assessment of the LGN volume using 7 MRI in the presence of central visual loss due to STGD.

## 2. Materials and Methods

### 2.1. Patients

The study was performed as a prospective, non-invasive, case–control study. Approval from the Ethics Committee of the Medical University of Lublin was obtained (KE-0254/257/12/2023). Prior to providing informed consent, all participants were informed of the purpose, methods, and potential risks. A total of 18 patients (mean age, 33 years; range, 17–54 years) were recruited at the Chair and Department of General and Pediatric Ophthalmology of the Medical University of Lublin, Poland (Table 1). Inclusion criteria were as follows: clinical diagnosis of STGD and age greater than 16 years. Exclusion criteria were as follows: intraocular pathologies that would affect visual acuity. The demographic characteristics of the study population are summarized in Table 1.

All patients underwent ophthalmologic examinations, including best-corrected visual acuity using Snellen charts, microperimetry (Maia), slit-lamp examination, wide-field fundus photography (Optos), optical coherence tomography (SD-OCT), and fundus autofluorescence. All STGD patients first underwent MRI 1.5 T to exclude other CNS pathologies. Additionally, 15 healthy patients (mean age, 31 years; range, 20–44 years) were also examined with MRI 7T. Inclusion criteria for the control group were as follows: best-corrected visual acuity of 20/20 and no history of previous ophthalmologic or neurologic disease. To examine whether age differed between the groups, a *t*-test was conducted. There was no significant difference in age between the groups. The *t*-test result, t = −0.76 (*p* = 0.45), was not significant. The assumptions of the test were verified using the Shapiro–Wilk test (normality) and Levene’s test (homogeneity of variance) (Table 2).

### 2.2. 7 Tesla MRI Data Acquisition

MRI data for this study were obtained at the ECOTECH COMPLEX (Lublin, Poland) using a Discovery MR950 7 T MRI system (GE Healthcare, Tokyo, Japan) with a gradient strength of 50 mT/m and a slew rate of 200 T/m/s. The coil configuration used for examinations was a two-channel birdcage coil driven in quadrature for transmission and a 32-channel array coil for reception (Nova Head 32-channel head coil, 2Tx/32Rx). The imaging protocol in the 7 T examination contained two sequences without contrast agent injection: 3D BRAVO T1-weighted and 3D MT-weighted SILENT obtained with parameters from Table 3.

### 2.3. Data Analysis

Analyses of the volume and cortical thickness of the brain were performed using the open-source automated software package FreeSurfer (version 7.4.1, Massachusetts General Hospital, Harvard Medical School; http://surfer.nmr.mgh.harvard.edu, accessed on 20 June 2023). We used “recon all” reconstruction, which reconstructs a two-dimensional cortical surface from a three-dimensional volume using a high-resolution T1-weighted anatomical scan with high contrast between the white matter and the grey matter. This procedure incorporates typical data reconstruction steps such as skull stripping, volumetric registration, normalization, volumetric labeling, segmentation, smoothing, and cortical parcellation with a voxel size of 1 mm^3^ (https://surfer.nmr.mgh.harvard.edu/fswiki/recon-all, accessed on 20 June 2023). Tabulated data summarizing the segmented results were gathered using a few FreeSurfer summarizing scripts. Volume assessment was performed in accordance with the data obtained from the Desikan–Killiany parcellation atlas. The LGN volume was calculated using ITK-SNAP version 4.0.0-rc.2, which allows manual delineation of anatomical regions of interest on 3D medical images. Histopathological assessment of the LGN size in the studied patients was impossible; therefore, we do not know the true value. Automatic segmentation of LGN volume measurements is unavailable because of the blurred borders, small size, and complex anatomy of the LGN. The 3D MT-weighted SILENT sequence was evaluated independently by three radiologists. One of them is also a Ph.D. student at the University of Technology (MM), and two of them are neuroradiologists (KW, MT). They applied the same linear image contrast adjustment: minimum 250, maximum 1750, level 1000, window 1500, and measured the LGN images in the same place, on the same computer (Advanced Workstation, GE), on the same calibrated monitor (BARCO, dedicated to the evaluation of medical radiology studies). All authors, including the three radiologists, participated in the development of measurement methods and the initial assessment of the 10-patient sample. This collaborative initial work was intended to ensure methodological uniformity of the measurement. A sample of a typical 3D MT-weighted SILENT scan of the brain with left and right 3D-LGN segmentations for control and Stargardt disease-affected brains is available under the link: www.dropbox.com/scl/fi/whz67omuydhsjjzlk69ut/LGN-Segmentation.zip?rlkey=mmniyqhxpb07x74iaandkqv81&e=3&st=in7mu6g9&dl=1, accessed on 20 June 2023. It was demonstrated that there were no significant differences between the measurements taken on both the left and right sides. A one-way ANOVA was conducted to compare the mean values of 1 LGN L, 2 LGN L, and 3 LGN L. The results showed no statistically significant differences between groups, F(2, 93) = 0.249, *p* = 0.78. A one-way ANOVA was conducted to compare the mean values of 1 LGN R, 2 LGN R, and 3 LGN R. The results showed no statistically significant differences between groups, F(2, 93) = 0.096, *p* = 0.909 (Table 4).

### 2.4. Statistical Analysis

All statistical analyses were performed in R (version 4.3.3; R Core Team, 2024). The following packages were used: dplyr, ggplot, tidyverse, ggpubr, flextable, and car. Data were processed using readxl and openxlsx, while statistical tests were conducted with the stats package (R Core Team, 2024). The level of significance was set at *p* = 0.05 for each analysis. To determine whether brain structures, particularly the LGN, differed between the control group and patients with STGD (hereinafter referred to as the study group), significance tests were conducted to compare these two groups. For two independent groups, Student’s *t*-test was used to compare the mean size of the left and right LGN between the study and control groups. Additionally, the LGN-to-thalamus ratio was analyzed and compared between the groups using the *t*-test to assess potential differences in relative LGN size. The assumptions of the parametric test were verified using the following tests: the normality of variable distribution in both groups was assessed using the Shapiro–Wilk test, and the homogeneity of variances was examined using Levene’s test. To assess whether the LGN and thalamus variables on the left and right sides, respectively, were correlated, a scatter plot was created, and Pearson’s correlation coefficient was calculated. The relationship between LGN volume and visual acuity, retinal sensitivity in microperimetry, and central retinal thickness in OCT was examined using both Pearson’s and Spearman’s correlation coefficients.

## 3. Results

LGN measurements were performed in 33 subjects (15 from the control group and 18 patients with Stargardt disease) (Table 5).

**Figure 1:**
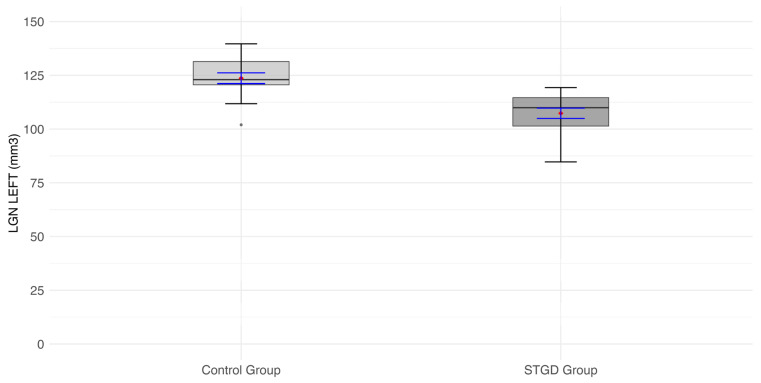
The box plot for the volume (mm^3^) of the left LGN in the studied group (patients with Stargardt disease—STGD) and the control group. Red diamonds indicate the mean values, while blue lines represent confidence intervals.

The mean LGN volume of the left hemisphere in the study group (107.4 ± [10.20] mm^3^) was significantly smaller than in the control group (123.6 ± [9.77] mm^3^; t(31) = 4.66, *p* < 0.0001) (Figure 1).

**Figure 2:**
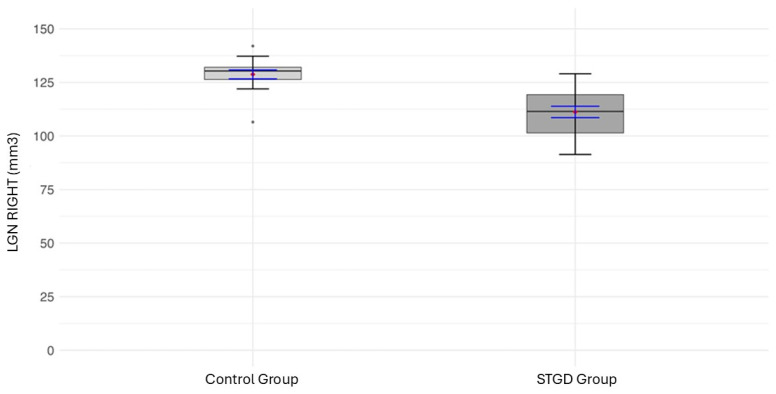
The box plot for the volume (mm^3^) of the right LGN in the studied group (patients with Stargardt disease—STGD) and the control group. Red diamonds indicate the mean values, while blue lines represent confidence intervals.

The mean LGN volume of the right hemisphere in the study group (111.2 ± [11.26] mm^3^) was significantly smaller than in the control group (128.7 ± [8.06] mm^3^; t(31) = 5.04, *p* < 0.0001) (Figure 2) (Table 6).

**Figure 3:**
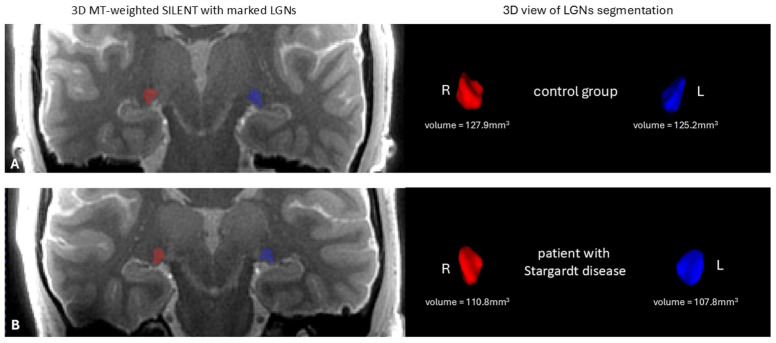
On the left—the anatomical image of the brain 3D MT-weighted SILENT with marked LGNs; on the right—3D view results of LGN manual segmentation using ITK-SNAP software, version 4.0.0-rc.2. Images show one case chosen from the control group (**A**) and one from patients with Stargardt disease (**B**). Images acquired at the Ecotech Complex (Lublin, Poland).

The anatomical image of the mean LGN in both hemispheres in a STGD patient and a patient from the control group is shown in Figure 3.

### 3.1. Association of Thalamus with LGN

It was investigated whether the LGN depends on the thalamus. Correlations between variables are insignificant, indicating no relationship between the left thalamus and left LGN, even in healthy volunteers in the control group. For the right hemisphere, the situation was analogous. There were no significant correlations between variables, and no dependencies were observed on the scatter plot.

### 3.2. LGN-to-Thalamus Ratio Analysis

The LGN-to-thalamus ratio was calculated to assess potential differences between the study and control groups. The following formula was used (Equation (1)):

(1) $$w_{lh}=\frac{{LGN}_{lh}}{{Thalamus}_{lh}},$$

Equation (1). Calculation of the left LGN-to-thalamus volume ratio, where ${LGN}_{lh}$ represents the volume of the left LGN, and ${Thalamus}_{lh}$ represents the volume of the left thalamus. This ratio (Equation (2)) provides a normalized measure of LGN volume relative to the thalamus, facilitating comparisons between groups.

(2) $$w_{rh}=\frac{{LGN}_{rh}}{{Thalamus}_{rh}},$$

Equation (2). Calculation of the right LGN-to-thalamus volume ratio, where ${LGN}_{rh}$ represents the volume of the right LGN, and ${Thalamus}_{rh}$ represents the volume of the right thalamus. This ratio provides a normalized measure of LGN volume relative to the thalamus, facilitating comparisons between groups.

**Figure 4:**
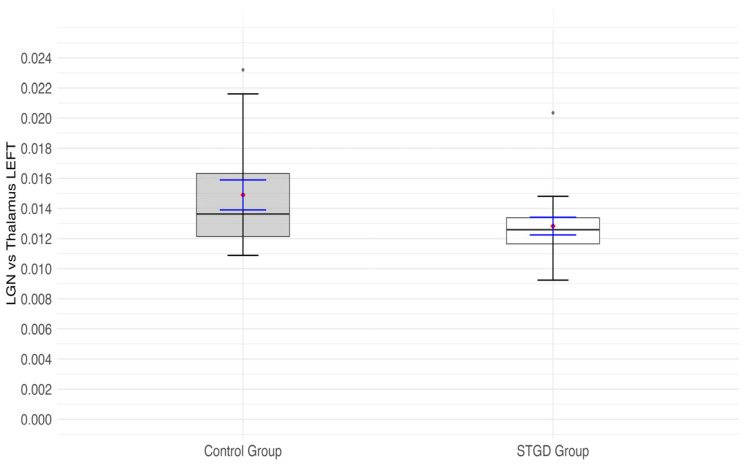
The box plot for the ratio of the left (lh) lateral geniculate nucleus (LGN) and left (lh) thalamus in the studied group (Stargardt disease—STGD) and control group. Red diamonds indicate the mean values, while blue lines represent confidence intervals.

The insignificant result of the Student’s *t*-test (t(31) = 1.84; *p* = 0.076) indicated that the ratio of LGN to thalamus in the left hemisphere was not significantly lower in the group of patients with STGD than in the control group (Figure 4).

**Figure 5:**
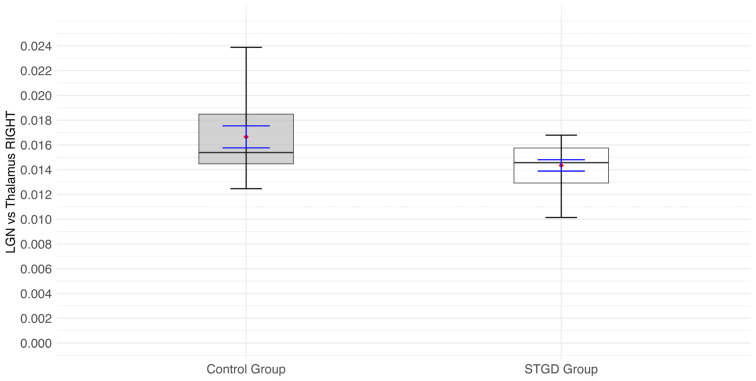
The box plot for the ratio of the right (rh) LGN and right (rh) thalamus in the study group (Stargardt disease—STGD) and control group. Red diamonds indicate the mean values, while blue lines represent confidence intervals.

A significant result of the Student’s *t*-test (t(31) = 2.38; *p* = 0.024) indicates that the ratio of LGN to thalamus in the right hemisphere was significantly lower in the group of patients than in the control group (Figure 5. In the study group, the right LGN was 0.014 of the right thalamus (in other words, the right LGN was 71 times smaller than the right thalamus), while in the control group, the right LGN was 0.017 of the right thalamus (in other words: the right LGN is 59 times smaller than the right thalamus).

Based on the scatter plot of LGN (left) and right eye visual acuity and retinal sensitivity in microperimetry, no clear linear or nonlinear trend was observed. This is supported by the calculated correlation coefficients, which were not statistically significant. Similarly, no significant relationship was found between LGN (right) and left eye visual acuity and central retinal thickness in OCT.

For the right LGN and visual acuity, the Pearson correlation coefficient was r = 0.24 (*p* = 0.335); for the left eye and visual acuity, the Pearson correlation coefficient was r = 0.645 (*p* = 0.645). For the right LGN and retinal sensitivity in microperimetry, the Pearson correlation coefficient was r = 0.30 (*p*= 0.221); for the left eye and retinal sensitivity in microperimetry, the Pearson correlation coefficient was r = –0.04 (*p*= 0.885). Moreover, no significant associations were found between LGN volume and central retinal thickness. For the right LGN, the Pearson correlation coefficient was r = 0.2122 (*p* = 0.398); for the left eye, the Pearson correlation coefficient was r =−0.1624 (*p* = 0.52). Spearman correlation coefficients were also not significant for all visual function and OCT parameters.

## 4. Discussion

This is the first study investigating the LGN in patients with STGD. MRI 7T using 3D MT-weighted SILENT sequence allows examination of the LGN in the visual pathway. Ultra-high-field imaging is an important step towards fully understanding the structure and function of the visual pathway. The precise quantitative assessment of the LGN volume was highly consistent thanks to the use of our custom 3D MT-W SILENT sequence in the 7T GE MR device and the cooperation of three experienced radiologists.

The first finding in our study was that LGN volume is smaller in patients with STGD than in the control group. Another finding of this investigation was that the average proportion of LGN to thalamus did not differ significantly between the study group and the control group. The precise determination of LGN in vivo is technically challenging due to its relatively small size and subcortical location [20].

Both anterograde and retrograde neurodegenerative cascades are proposed to occur through synapses in the LGN, but the LGN itself was not part of previous studies because of the technical difficulty of its measurement [21]. Up to now, analysis of absolute LGN measurements has not accounted for the influence of the total size of the brain and its atrophy or forms of degeneration. Additionally, the measurements were influenced by the indistinct outline of the LGN. Reference to the very clearly demarcated, large subcortical structure of the thalamus was an attempt to objectify the measurements. The LGN/thalamus ratio used in this study allowed independence from the dimensions of the patient, and especially the brain.

In the literature, values of normal LGN volume vary considerably when measured using different magnetic field strengths of 1.5, 3, and 7 Tesla MRI [22], ranging from 76 mm^3^ [21] to 267 mm^3^ [23], with the average of the left and right LGN volumes being 149.6 ± 45.8 mm^3^. Thus, in our study, the volumes of the right and left LGN were lower than this average. However, different age groups, imaging protocols, and delineation methods have been used in different studies [22]. LGN volumes measured on 7T systems are generally lower than those measured using 3T MRI units [24,25]. The explanation may be that the magno- and parvocellular layers of the LGN both contain ferric ion, and even a minute amount of any ferromagnetic substance causes a reduction in magnetic resonance signal intensity, which is much higher at higher magnetic fields [26].

Gupta and colleagues reported that the LGN height was significantly decreased in patients with glaucoma [27]; this decrease was also shown by coronal proton density MR imaging [28] and with 7 Tesla MRI in normal-tension glaucoma [29]. Similarly, LGN volume reduction has been seen in patients with postgeniculate lesions using 3 Tesla MRI. LGN volume decrease correlated with ganglion cell layer thickness reduction as a sign of trans-synaptic retrograde neuronal degeneration [30]. The LGN volume has also been found to be altered in ophthalmologic and neurodegenerative pathologies of afferent and efferent visual systems, such as albinism, amblyopia, and Leber hereditary optic neuropathy [19,31,32]. Moreover, decreased LGN volume has been observed in multiple sclerosis [33], and it is anticipated that it may be a promising marker reflecting damage to the visual pathway. A specific assessment of the LGN is crucial to investigate neurodegeneration occurring specifically in the visual pathway. LGN has been found to be decreased in twelve patients suffering from RP due to RPGR pathogenic variants [34].

It seems that a decreased volume of the LGN in patients with STGD may represent an adaptive process in response to decreased central visual acuity. It indicates a profound effect of ocular disease involving the macula on the LGN structures.

Likewise, it has already been proven that retinal gene therapy in retinal dystrophies promotes robust expansion and an increase in LGN volume. Ashtari and coworkers examined seven patients with Leber congenital amaurosis (LCA), another retinal dystrophy, by functional MRI [35]. The response to visual stimulation through untreated eyes of LCA patients showed heightened fMRI responses in the superior colliculus and diminished activities in the LGN. After gene therapy, stimuli presented to the treated eye elicited significantly stronger fMRI responses in the LGN and primary visual cortex. The authors concluded that this indicates some re-engagement of the geniculostriate pathway after gene therapy [36]. However, four of seven examined patients were children with a possibly higher range of brain plasticity. Moreover, patients with LCA had visual acuity worse than Snellen 20/160 or a visual field less than 20°.

Currently, there is no effective treatment for STGT; however, there are many attempts focused on research into novel therapeutic strategies, including gene therapy, stem cell therapy, and pharmacological interventions [37]. These treatments have given hope to the low-vision patient population, but it may take years before patients with genetic forms of retinal degenerative disease can benefit from such advances [38,39]. Moreover, there are difficulties in assessing the efficacy of these methods in patients with IRDs.

To date, functional 3 Tesla MRI has been used in patients with central scotoma due to macular diseases. In a study of two adult subjects with extensive bilateral central retinal lesions, parts of the visual cortex (including the primary visual cortex) that normally responded only to central visual stimuli were strongly activated by peripheral stimuli. Such activation was not observed with visual stimuli presented to the position of the former fovea and in control subjects with visual stimuli presented to corresponding parts of the peripheral retina [40].

In STGD, an absolute scotoma prevents foveal fixation; thus, the patient uses an eccentric location on the retina to fixate by directing their gaze away from the target. With time, most patients establish one specific eccentric retinal area as a kind of pseudo-fovea, called the “preferred retinal locus” (PRL) [41]. In a study of fMRI in two patients with central scotoma due to different macular degenerations, the patient with an established preferred retinal locus (PRL) exhibited significantly higher activation in the early visual cortex during the visual search task, especially during trials when the target stimuli fell in the vicinity of the PRL. Compared with those with less stable fixation, patients with stable eccentric fixation at the PRL exhibited greater performance levels and more brain activation. There were also other studies on fMRI in macular degenerations without retinal function examinations and with no genotype examinations [42,43]. In a more recent study of 24 STGD patients, Melillo and colleagues [44] showed stronger primary visual cortex (PVC) activation in STGD1 patients with a more preserved retinal function and macular structure. We already know that approximately 50% of the PVC is devoted to the central 15° of the visual field.

Our study showed no relationship between LGN volume and visual function (visual acuity and microperimetry), or structural examination of the retina (central retinal thickness in OCT). Thus, it does not clarify the clinical significance of the reduction in LGN volume in STGD patients. Possibly, RNFL thickness should be measured in STGD patients, as in studies regarding optic nerve diseases and 7 Tesla MRI. In a study of LGN in glaucoma, there were no significant correlations between visual field indices and LGN volumes in early and advanced glaucoma groups. Significant correlations between mean retinal nerve fiber layer thickness and LGN volume were observed for the control group and early glaucoma, but not for advanced glaucoma [29]. In Leber hereditary optic neuropathy patients, the volume of the right LGN was strongly correlated with the averaged thickness value of the right retinal nerve fiber layer [32].

The shortcoming of our study is the relatively small number of patients included. The small sample size may introduce selection bias, limiting the generalizability of the study results to a broader population. Thus, a larger study would be useful to assess LGN morphometry in STGD patients. Moreover, the present study employed manual segmentation of LGN volume. Although three radiologists followed the same standards, manual segmentation is highly dependent on the subjective judgment of the operator, making it difficult to completely avoid inter-individual differences and biases. Even with the same image contrast adjustment parameters, measurement equipment, and calibrated displays, variations in visual perception and interpretation among different doctors may still lead to measurement differences. For instance, for LGN regions with unclear boundaries or complex structures, doctors may disagree on determining the boundaries, which can affect the reliability of the measurement results. Additionally, prolonged manual segmentation may cause fatigue in radiologists, potentially reducing measurement accuracy.

The primary pathology in STGD is retinal, but neuroplastic changes in the brain can occur over time. Further studies are needed to assess, with 7 Tesla MRI, other parts of the visual pathway and remaining parts of the brain, including the cortex. Understanding the plastic changes in the brain in response to visual field defects is essential for any attempt to develop efficient rehabilitation strategies in hereditary retinal dystrophies. It is also an example of how genetically conditioned disorders compromise proper structural and functional development in the brain. It is important to develop new tools and rehabilitation strategies to help maximize the use of residual visual functions. Maximal use of residual visual capabilities may be the key to maintaining experience-dependent plasticity of the visual circuits until retinal therapeutic interventions become available. Moreover, it is crucial to determine to what extent any changes reverse their course following genetic therapy, leading to partial visual recovery.

We suppose that a new understanding of central visual processing in inherited retinal diseases can facilitate future strategies to augment vision. We also hope that quantitative volumetric assessment of the LGN will enable future longitudinal studies to gain insight into the dynamics and extent of LGN atrophy in relation to retinal dystrophies. We intend to expand in the future the number of patients with STGD examined with 7 Tesla MRI and to compare the values of LGN with results obtained in patients with other retinal dystrophies.

## 5. Conclusions

LGN volume in MRI 7T imaging is reduced in patients with STGD. MRI 7T using the 3D MT-weighted SILENT sequence provides new insight into structural changes in the brain in retinal dystrophies and offers a new possible marker of the response to future therapies in STGD.

## Figures and Tables

**Table 1:** Demographic data and visual acuity of patients with Stargardt disease included in the study.

Patient ID	Age (Years)	Gender (F: Female; M: Male)	Visual Acuity Right Eye (Snellen)	Visual Acuity Left Eye (Snellen)
1	44	F	0.05	0.05
2	54	F	0.02	0.02
3	19	M	0.02	0.02
4	42	M	0.01	0.01
5	21	M	0.05	0.05
6	43	M	0.05	0.05
7	20	M	0.01	0.01
8	48	M	0.05	0.05
9	33	M	0.02	0.02
10	34	F	0.1	0.1
11	38	M	0.05	0.01
12	49	M	0.05	0.05
13	33	F	0.05	0.05
14	31	F	0.05	0.05
15	32	M	0.05	0.05
16	17	M	0.05	0.05
17	23	F	0.2	0.1
18	18	M	0.1	0.1

**Table 2:** Comparison of age between Stargardt disease and control group.

	Mean	Standard Deviation	Levene’s Test		Shapiro–Wilk		*t*-Test	
			F	*p*	W	*p*	t	*p* Value
Age Stargardt disease	33.3	11.74	3.165	0.0851	0.94	0.2639	0.76	0.4505
Age control group	30.7	7.15			0.95	0.6035		

**Table 3:** Protocols used in the present study for 7 Tesla MRI imaging of the brain. Abbreviations: FOV (field of view), TE (echo time), TR (repetition time), TI (inversion time), and NEX (number of excitations).

	3D BRAVO T1-W	3D MT-W SILENT
Scan duration	4 min 24 s	6 min 30 s
FOV (cm)	22 × 22	17.6 × 17.6
Slice thickness [mm]	1.0	0.8
TE [ms]	2.6	0.0
TR [ms]	6.6	257
TI [ms]	450	not applicable
Matrix size	288 × 288	224 × 224
NEX	1	3
Flip Angle	12	2

**Table 4:** Descriptive statistics of LGN measurements taken by three radiologists.

Variable	Mean	SD	Median	Min	Max	N
1 LGN L	114.12	13.02	115.70	87.00	141.00	33
2 LGN L	116.28	12.89	118.00	88.90	140.00	33
3 LGN L	114.71	13.45	116.50	78.27	138.00	33
1 LGN R	119.23	13.65	120.00	91.90	142.60	33
2 LGN R	118.45	13.44	123.30	90.70	140.70	33
3 LGN R	120.02	13.27	124.40	91.51	143.30	33

**Table 5:** Absolute volumes of LGN for all 33 subjects (15 from the control group and 18 patients with Stargardt disease).

Control Group					Patients with Stargardt Disease				
No	Age	Gender	LGN LEFT	LGN RIGHT	No	Age	Gender	LGN LEFT	LGN RIGHT
1	33	M	133.67	134.53	1	44	F	95.84	107.13
2	22	M	132.10	137.23	2	54	F	95.27	99.57
3	24	M	130.67	132.23	3	19	M	84.72	95.89
4	27	M	112.77	122.27	4	42	M	114.77	109.77
5	27	M	126.53	126.67	5	21	M	114.20	112.07
6	28	M	111.80	121.97	6	43	M	112.57	129.03
7	24	M	139.67	141.97	7	20	M	105.43	97.31
8	28	M	133.40	130.17	8	48	M	100.17	99.00
9	20	M	124.40	127.83	9	33	M	116.80	127.87
10	33	M	121.50	126.03	10	34	F	105.07	107.03
11	37	F	123.00	130.33	11	38	M	119.30	120.87
12	36	F	101.98	106.50	12	49	M	110.10	123.30
13	39	F	120.27	130.93	13	33	F	109.30	110.83
14	44	F	122.00	131.83	14	31	F	90.36	91.37
15	39	F	120.90	130.57	15	32	M	117.87	116.77
					16	17	M	112.73	117.00
					17	23	F	109.80	117.07
					18	18	M	117.93	120.03

**Table 6:** Comparison of LGN size between the study group (S) and control group (C).

	Gr.	Mean	SD	Median	Levene’s Test		*t*-test		95 Percent Confidence Interval	
					F	*p*	*t* (df)	*p* Value	Left	Right
LGN LEFT	S	107.4	10.20	110.0	0.05	0.83	4.66; (31)	<0.001	9.16	23.44
	C	123.6	9.77	123.0						
LGN RIGHT	S	111.2	11.26	111.5	3.54	0.07	5.04; (31)	<0.001	10.43	24.61
	C	128.7	8.06	130.3						

## Data Availability

The datasets analyzed during the current study may be available from the corresponding author upon reasonable request.
